# A Case of Sporadic Creutzfeldt-Jakob Disease Presenting as Conversion Disorder

**DOI:** 10.1155/2017/2735329

**Published:** 2017-05-03

**Authors:** Nikhil Yegya-Raman, Rehan Aziz, Daniel Schneider, Anthony Tobia, Megan Leitch, Onyi Nwobi

**Affiliations:** ^1^Rutgers-Robert Wood Johnson Medical School, Piscataway, NJ, USA; ^2^Psychiatry at Rutgers-Robert Wood Johnson Medical School, Piscataway, NJ, USA; ^3^Neurology at Rutgers-Robert Wood Johnson Medical School, Piscataway, NJ, USA

## Abstract

*Background*. Creutzfeldt-Jakob disease is a rare disorder of the central nervous system. Its initial diagnosis may be obscured by its variable presentation. This case report illustrates the complexity of diagnosing this disease early in the clinical course, especially when the initial symptoms may be psychiatric. It offers a brief review of the literature and reinforces a role for consultation psychiatry services.* Methods*. PUBMED/MEDLINE was searched using the terms “Creutzfeldt-Jakob disease”, “psychiatric symptoms”, “conversion disorder”, “somatic symptom disorder”, “functional movement disorder”, and “functional neurologic disorder”.* Case*. The patient was a 64-year-old woman with no prior psychiatric history who was initially diagnosed with conversion disorder and unspecified anxiety disorder but soon thereafter was discovered to have Creutzfeldt-Jakob disease.* Discussion*. This case highlights the central role of psychiatric symptoms in early presentations of Creutzfeldt-Jakob disease. Still, few other cases in the literature report functional neurological symptoms as an initial sign. The consultation psychiatrist must remain alert to changing clinical symptoms, especially with uncharacteristic disease presentations.

## 1. Introduction

Creutzfeldt-Jakob disease (CJD) is a rapidly progressive, fatal neurodegenerative disease caused by aggregation of misfolded prion proteins. A prion, named by Prusiner [1], is a proteinaceous infectious agent. It is abundant in healthy neurons as a soluble protein. However, it can be converted to a *β*-pleated form, PrP^Sc^ (prion protein scrapie), which can form insoluble aggregates in the nervous tissue. These are resistant to degradation by proteases, can convert soluble prion proteins to PrP^Sc^, and are transmissible. PrP^Sc^ deposition leads to cortical and subcortical loss in the absence of inflammation, causing small vacuoles and a spongiform appearance [2]. The distribution of spongiform changes in the brain likely leads to the diverse neurological and psychiatric manifestations associated with this condition.

There are four forms of CJD: sporadic, variant, familial, and iatrogenic [3]. Sporadic CJD is caused by spontaneous prion protein transformation or somatic gene mutations. Variant CJD arises after ingestion of meat products derived from animals afflicted with bovine spongiform encephalopathy (BSE), also known as “mad cow disease.” Familial CJD results from autosomal dominant mutations of PRNP, the prion protein gene. Related conditions involving PRNP mutations include fatal familial insomnia and Gerstmann-Straussler-Scheinker syndrome. Although generally controlled by modern practices, iatrogenic CJD has been reported to occur after administration of cadaveric human pituitary hormones, from contaminated neurosurgical instruments, and following corneal or dural graft transplants [2, 3].

The sporadic form (sCJD) constitutes 85% of cases. It typically presents in the sixth decade, affects males and females equally, and has an incidence of approximately one case per million persons per year [4]. The classic triad of CJD is rapidly progressive dementia, myoclonus, and ataxia. Additional signs include behavioral dysfunction, dysphasia, pyramidal or extrapyramidal signs, cortical blindness, and primitive reflexes [3]. Rigidity, myoclonus, and characteristic electroencephalogram (EEG) complexes often present late [5]. Most patients decline rapidly to a state of akinetic mutism. The mean duration of illness is about 4.5 months, and 80% die within one year [3].

Psychiatric symptoms are more common in sCJD than often suspected. They occur in 80–90% of patients over the course of their illness [6–8], and 20–26% of patients present initially with psychiatric symptoms. They arise early and can consist of sleep disturbance, psychosis, depression, cognitive impairment, agitation, or irritability. There have been several case reports of sCJD presenting with psychiatric signs [2, 9–11]. Given the high comorbidity of sCJD with psychiatric findings, sCJD may be more appropriately classified as a neuropsychiatric disorder [6].

Turning to movement disorders, they are reported in a significant number of patients with CJD. Myoclonus is the most frequent finding, but dystonia, choreoathetosis, tremor, hemiballismus, and atypical parkinsonism have also been noted [12]. Functional neurological symptoms or conversion disorder seem to have been described in only two case reports [5, 11].

There are three levels of diagnostic certainty of sCJD: definite, probable, and possible (Table 1). Definite diagnosis of CJD relies on neuropathology or on brain biopsy by demonstration of PrP^Sc^ in the nervous tissue. Probable and possible diagnoses rely on a combination of neurological signs, specific EEG findings, cerebrospinal fluid (CSF) biomarkers, and distinct magnetic resonance imaging (MRI) lesion patterns [13].

Because sCJD is rare, presents with nonspecific symptoms, and is difficult to definitely diagnose, practitioners must maintain a broad differential diagnosis at the onset. This includes, but is not limited to, neurodegenerative, vascular, autoimmune, infectious, toxic/metabolic, metastatic, and iatrogenic etiologies [14]. We present a case of sCJD in which the initial diagnostic workup ruled out an array of known causes, including CJD. We discuss the challenge this patient posed to the care team, along with the role of the consultation psychiatrist.

## 2. Case

Ms. T was a 64-year-old Caucasian woman with no past psychiatric history and a past medical history of hypertension, hypothyroidism, and Lyme disease who presented with two months of new-onset worsening tremulous jerky movements of her right upper extremity, right-sided numbness, ataxia, headaches, and joint pain. The patient did not exhibit myoclonus or confusion. Her symptoms had occurred in the context of several psychosocial stressors. Ms. T was admitted to a general inpatient medical unit for further evaluation and treatment.

Prior to her hospitalization, she had been taking levothyroxine for hypothyroidism and ramipril for hypertension. There was no history of head injury, surgeries, or seizures. She denied a history of alcohol or illicit substance use disorders and was a nonsmoker.

Psychiatry was consulted within the first week of her hospitalization. Although she did not have a psychiatric history, the patient had been facing significant life stressors. She was divorced, lived alone, and had few confidants. One year prior, she had lost her mother and another close family member. In order to tend to her ailing mother, she had taken an early retirement from a job in healthcare and relocated to reside with her. She now wished to return to her employment. Nevertheless, apart from anxiety related to her health, the patient denied all psychiatric symptoms, including depression.

During the first week of hospitalization, the neurology team observed that her tremors and other neurological symptoms were distractible, variable, and entrainable. Physical examination was notable for an intermittent resting tremor of the right arm and leg, along with decreased muscle strength (4/5) in the right arm and leg. Deep tendon reflexes were 2+ and symmetric throughout. Toes were mute bilaterally. She had decreased right facial sensation, but no other abnormalities on cranial nerve testing. Gait was ataxic, but difficult to assess because her right lower extremity weakness affected her effort. On the Montreal Cognitive Assessment, she scored 22/30, losing points for recall of 0/5 items, inability to perform more than two serial 7's, and inability to repeat numbers backwards.

An extensive workup was performed. This included brain and cervical spine MRIs, chest and abdominal computerized tomography scans, serial EEGs, blood and CSF Lyme titers, herpes simplex virus titers, CSF 14-3-3 levels, lupus anticoagulant levels, and a paraneoplastic panel. Other teams apart from psychiatry and neurology were also consulted, including endocrinology, infectious disease, and rheumatology. Besides a mildly elevated thyroid-stimulating hormone (TSH), these initial efforts failed to reveal a possible etiology for her symptoms.

By the end of the first week of her hospitalization, the patient was given a provisional diagnosis of a functional neurological (movement) disorder by the neurology service. Given her presentation, in conjunction with an unremarkable workup, this diagnosis was maintained by the psychiatry team. Though the patient denied symptoms of depression, it was posited that her significant psychosocial stressors could be contributing to her symptoms. Escitalopram was recommended as a low risk intervention for both anxiety and possible depression; the patient provided informed refusal. Her hypothyroidism and mildly elevated TSH were addressed by adjusting her levothyroxine dosage. Physical and occupational therapy were started, with the intention of addressing her functional disabilities.

After two weeks of hospitalization, the patient remained ill. Since her presentation was thought to be mainly functional, many consultants became less involved in her care. Then, her condition further declined. She started exhibiting expressive aphasia, refusing to eat or drink, and appearing markedly confused. She also began maintaining a rigid posture, with bilateral upper extremity tremors and myoclonus. A trial of high dose steroids, for a possible autoimmune or inflammatory condition, followed by a trial of benzodiazepines, for possible catatonia, did not change her state. Given her history of previously treated Lyme disease, her family believed she had developed Lyme neuroborreliosis. Later, she was treated empirically for neuroborreliosis with IV antibiotics without any improvement.

Three weeks into her hospital stay, a repeat 24-hour EEG showed focal slowing over the frontotemporal/central region, which signified a change from her original EEG. In light of this, the psychiatry team advocated for specialist reconsultation and further medical/neurological workup, including repeat testing.

Indeed, four weeks into her hospital stay, a repeat MRI showed new cortical T2 fluid attenuation inversion recovery (FLAIR) hyperintensities, a repeat 24-hour EEG showed triphasic waves, and neuron specific enolase (NSE) levels, which had not been measured previously, were markedly elevated. Clinical suspicion for sCJD was high, despite only meeting the criteria for “possible” sCJD. Ultimately, a definitive diagnosis of sCJD was made via brain biopsy. The patient was eventually transferred to hospice care and passed away two months after her initial arrival to the hospital or four months after symptom onset.

## 3. Discussion

Based on the Centers for Disease Control and Prevention's criteria for the diagnosis of sCJD, Ms. T exhibited cerebellar signs (ataxia) and extrapyramidal signs (tremors) at the onset, which, when interpreted in the context of impaired neurocognitive testing and an unremarkable diagnostic workup, arguably placed her in the “possible” sCJD category. The initial workup was extensive but only included CSF 14-3-3 levels. Other markers such as CSF Tau, S100b, and NSE could have aided. The sensitivities of these markers range from 73% to 86% [15]. Newer tests, such as real-time quaking-induced conversion (RT-QuIC) assays of CSF and nasal mucosa, have a diagnostic sensitivity and specificity of 96% and 100%, respectively [16]. Although there are no current disease-modifying therapies for sCJD, prompt diagnosis allows for appropriate palliative care to be initiated, provides some closure to the patient and the patient's family, and reduces the risk of iatrogenic transmission.

It remains unclear whether the patient's functional symptoms were an early manifestation of sCJD or a cooccurring phenomenon. Functional symptoms have been found in the initial stages of many neurological disorders [17]. Perhaps early prion mediated neuronal loss in particular brain regions led to our patient's early symptoms, which were consistent with a functional neurological disorder. As the disease progressed, they could have resolved into clearer and more distinct neurological signs. If so, this could challenge our understanding of the mechanism behind functional illness and the binary distinction of mind versus body. Conceivably, subtle neurological changes may underlie pathological changes which eventually result in the phenotype of functional symptoms.

This case also illustrates the difficulty encountered when patients are assigned a functional or conversion disorder diagnosis. As such, it was not that the provisional diagnosis of a functional disorder was incorrect but that it produced an assumption that there were or could not be other factors at play. All of the patient's symptoms began to be viewed as functional, and the diagnosis became immutable. When the patient's presentation changed, the psychiatry team, which had remained involved, suspecting that something was afoot, recommended additional diagnostic testing and specialist reconsultation. Thus, the diagnosis of conversion disorder can lead practitioners to foreclose on other possibilities, and it may be up to the psychiatrist to reopen them.

Overall, sCJD is a rapidly progressive, fatal neurodegenerative disease that can present in a variety of ways and is difficult to diagnose in the early stages. This case highlights the need for clinical vigilance in first and/or atypical presentations of mental illness. This is imperative perhaps more so in cases which appear to be the result of a functional disturbance. Repeated assessments and diagnostic testing may be required. As many patients with sCJD exhibit psychiatric symptoms early in their course, sCJD should remain on the differential diagnosis whenever a patient presents with rapidly progressing neurological symptoms, even when initial diagnostic testing is unremarkable and there seems to be an underlying emotional component. This case challenges our perception of conversion disorder as an entirely psychological phenomenon and neurological disease as entirely biological [5]. If disease can exist in an immaterial, “functional” realm, how then can it influence a brain made of matter and mass and produce bodily symptoms? Perhaps the connections are subtler than thought.

## Figures and Tables

**Table 1:** Centers for disease control and prevention's diagnostic criteria for sCJD^*∗*^.

A	B	C
(1) Myoclonus (2) Visual or cerebellar signs (3) Pyramidal/extrapyramidal signs (4) Akinetic mutism	(1) Periodic sharp wave complexes on EEG during an illness of any duration (2) Positive 14-3-3 CSF assay in patients with a disease duration of less than 2 years (3) MRI high signal abnormalities in caudate nucleus and/or putamen on diffusion weighted or fluid attenuation inversion recovery imaging	Routine investigations that do not indicate an alternative diagnosis

^*∗*^Definite sCJD = diagnosed by standard neuropathological techniques; and/or immunocytochemically and/or Western blot confirmed protease-resistant PrP and/or presence of scrapie-associated fibrils.

Probable sCJD = rapidly progressive dementia + at least 2 of (A) + at least 1 of (B) + (C).

Possible sCJD = progressive dementia with duration of illness less than 2 years + at least 2 of (A) + none of (B) + (C).
