# Corrigendum to “A Case of Sporadic Creutzfeldt-Jakob Disease Presenting as Conversion Disorder”

**DOI:** 10.1155/2018/3826863

**Published:** 2018-02-21

**Authors:** Nikhil Yegya-Raman, Rehan Aziz, Daniel Schneider, Anthony Tobia, Megan Leitch, Onyi Nwobi

**Affiliations:** ^1^Rutgers-Robert Wood Johnson Medical School, Piscataway, NJ, USA; ^2^Psychiatry at Rutgers-Robert Wood Johnson Medical School, Piscataway, NJ, USA; ^3^Neurology at Rutgers-Robert Wood Johnson Medical School, Piscataway, NJ, USA

In the Introduction of the article titled “A Case of Sporadic Creutzfeldt-Jakob Disease Presenting as Conversion Disorder” [1], the text reading “Creutzfeldt-Jakob disease (CJD) is a rapidly progressive, fatal neurodegenerative disease caused by aggregation of misfolded prion proteins. A prion, named by Prusiner [1], is a proteinaceous infectious agent. It is abundant in healthy neurons as a soluble protein. However, it can be converted to a *β*-pleated form, PrP^Sc^ (prion protein scrapie), which can form insoluble aggregates in the nervous tissue. These are resistant to degradation by proteases, can convert soluble prion proteins to PrP^Sc^, and are transmissible. PrP^Sc^ deposition leads to cortical and subcortical loss in the absence of inflammation, causing small vacuoles and a spongiform appearance [2]. The distribution of spongiform changes in the brain likely leads to the diverse neurological and psychiatric manifestations associated with this condition.

There are four forms of CJD: sporadic, variant, familial, and iatrogenic [3]. Sporadic CJD is caused by spontaneous prion protein transformation or somatic gene mutations. Variant CJD arises after ingestion of meat products derived from animals afflicted with bovine spongiform encephalopathy (BSE), also known as “mad cow disease.” Familial CJD results from autosomal dominant mutations of PRNP, the prion protein gene. Related conditions involving PRNP mutations include fatal familial insomnia and Gerstmann-Straussler-Scheinker syndrome. Although generally controlled by modern practices, iatrogenic CJD has been reported to occur after administration of cadaveric human pituitary hormones, from contaminated neurosurgical instruments, and following corneal or dural graft transplants [2, 3].

The sporadic form (sCJD) constitutes 85% of cases. It typically presents in the sixth decade, affects males and females equally, and has an incidence of approximately one case per million persons per year [4]. The classic triad of CJD is rapidly progressive dementia, myoclonus, and ataxia. Additional signs include behavioral dysfunction, dysphasia, pyramidal or extrapyramidal signs, cortical blindness, and primitive reflexes [3]. Rigidity, myoclonus, and characteristic electroencephalogram (EEG) complexes often present late [5]. Most patients decline rapidly to a state of akinetic mutism. The mean duration of illness is about 4.5 months, and 80% die within one year [3]” should be updated as follows.

“Creutzfeldt-Jakob disease (CJD) is a rapidly progressive, lethal neurodegenerative disease caused by aggregation of misfolded prion proteins. A prion, named by Prusiner [1], is a proteinaceous infectious agent. It is found in healthy neurons as a soluble protein. However, it can be altered to a *β*-pleated form, PrP^Sc^ (prion protein scrapie), which can form insoluble aggregates in the nervous tissue. These are resistant to degradation by proteases, can transform soluble prion proteins to PrP^Sc^, and are communicable. PrP^Sc^ deposition leads to cortical and subcortical loss in the absence of inflammation. This gives rise to small vacuoles and a spongiform appearance. Ultimately, the scattering of spongiform changes likely produces the various neurological and psychiatric manifestations associated with this ailment [2].

There are four forms of CJD: sporadic, variant, familial, and iatrogenic [3]. Sporadic CJD is caused by spontaneous prion protein transformation or somatic gene mutations. Variant CJD arises after ingestion of meat products from animals afflicted with bovine spongiform encephalopathy (BSE), also known as “mad cow disease.” Familial CJD results from autosomal dominant mutations of PRNP, the prion protein gene. Related conditions involving PRNP mutations include fatal familial insomnia and Gerstmann-Straussler-Scheinker syndrome [2]. Although normally controlled by modern practices, iatrogenic CJD has been described to occur after administration of cadaveric human pituitary hormones, from contaminated neurosurgical instruments, and following corneal or dural graft transplants [2, 3].

The sporadic form (sCJD) typically presents in the sixth decade. It impacts men and women equally and constitutes 85% of cases. The incidence is approximately one case per million persons per year. The classic triad of CJD is rapidly progressive dementia, myoclonus, and ataxia. Additional signs include behavioral dysfunction, dysphasia, pyramidal or extrapyramidal signs, cortical blindness, and primitive reflexes [3]. Rigidity, myoclonus, and characteristic electroencephalogram (EEG) complexes frequently arise late [4]. Most patients deteriorate swiftly to a state of akinetic mutism. The mean duration of illness is around 4.5 months [3]. The highest mortality occurs in the 60–79-year-old age group [5], and 80% perish within one year [3].”

Accordingly, references 4 and 5 should be reversed as follows:

[4] H. B. Solvason, B. Harris, P. Zeifert, B. H. Flores, and C. Hayward, “Psychological versus biological clinical interpretation: a patient with prion disease,”* American Journal of Psychiatry*, vol. 159, no. 4, pp. 528–537, 2002.

[5] A. Ladogana, M. Puopolo, E. A. Croes et al., “Mortality from Creutzfeldt-Jakob disease and related disorders in Europe, Australia, and Canada,”* Neurology*, vol. 64, no. 9, pp. 1586–1591, 2005.

Additionally, the in-text citation of these references should be corrected as follows:

In the Introduction, the sentence “Functional neurological symptoms or conversion disorder seem to have been described in only two case reports [5, 11]” should be changed to “Functional neurological symptoms or conversion disorder seems to have been described in only two case reports [4, 11].”

In the Discussion, the sentence “This case challenges our perception of conversion disorder as an entirely psychological phenomenon and neurological disease as entirely biological [5]” should be changed to “This case challenges our perception of conversion disorder as an entirely psychological phenomenon and neurological disease as entirely biological [4].”
